# Identification of Seroreactive Proteins of *Leptospira interrogans* Serovar Copenhageni Using a High-Density Protein Microarray Approach

**DOI:** 10.1371/journal.pntd.0002499

**Published:** 2013-10-17

**Authors:** Carolina Lessa-Aquino, Camila Borges Rodrigues, Jozelyn Pablo, Rie Sasaki, Algis Jasinskas, Li Liang, Elsio A. Wunder, Guilherme S. Ribeiro, Adam Vigil, Ricardo Galler, Douglas Molina, Xiaowu Liang, Mitermayer G. Reis, Albert I. Ko, Marco Alberto Medeiros, Philip L. Felgner

**Affiliations:** 1 Bio-Manguinhos, Oswaldo Cruz Foundation, Brazilian Ministry of Health, Rio de Janeiro, Brazil; 2 Department of Medicine, Division of Infectious Disease, University of California Irvine, Irvine, California, United States of America; 3 Antigen Discovery Inc, Irvine, California, United States of America; 4 Department of Epidemiology of Microbial Diseases, Yale University, New Haven, Connecticut, United States of America; 5 Gonçalo Moniz Research Institute, Oswaldo Cruz Foundation, Brazilian Ministry of Health, Salvador, Brazil; 6 Institute of Collective Health, Federal University of Bahia, Salvador, Brazil; 7 Oswaldo Cruz Institute, Oswaldo Cruz Foundation, Brazilian Ministry of Health, Rio de Janeiro, Brazil; University of Tennessee, United States of America

## Abstract

**Background:**

Leptospirosis is a widespread zoonotic disease worldwide. The lack of an adequate laboratory test is a major barrier for diagnosis, especially during the early stages of illness, when antibiotic therapy is most effective. Therefore, there is a critical need for an efficient diagnostic test for this life threatening disease.

**Methodology:**

In order to identify new targets that could be used as diagnostic makers for leptopirosis, we constructed a protein microarray chip comprising 61% of *Leptospira interrogans* proteome and investigated the IgG response from 274 individuals, including 80 acute-phase, 80 convalescent-phase patients and 114 healthy control subjects from regions with endemic, high endemic, and no endemic transmission of leptospirosis. A nitrocellulose line blot assay was performed to validate the accuracy of the protein microarray results.

**Principal findings:**

We found 16 antigens that can discriminate between acute cases and healthy individuals from a region with high endemic transmission of leptospirosis, and 18 antigens that distinguish convalescent cases. Some of the antigens identified in this study, such as LipL32, the non-identical domains of the Lig proteins, GroEL, and Loa22 are already known to be recognized by sera from human patients, thus serving as proof-of-concept for the serodiagnostic antigen discovery approach. Several novel antigens were identified, including the hypothetical protein LIC10215 which showed good sensitivity and specificity rates for both acute- and convalescent-phase patients.

**Conclusions:**

Our study is the first large-scale evaluation of immunodominant antigens associated with naturally acquired leptospiral infection, and novel as well as known serodiagnostic leptospiral antigens that are recognized by antibodies in the sera of leptospirosis cases were identified. The novel antigens identified here may have potential use in both the development of new tests and the improvement of currently available assays for diagnosing this neglected tropical disease. Further research is needed to assess the utility of these antigens in more deployable diagnostic platforms.

## Author Summary

Leptospirosis is an infectious zoonotic disease that causes non-specific signs and symptoms in humans, which hampers the clinical diagnosis and treatment by physicians. Complications can occur if the proper treatment is not initiated early in the course of illness. Although the early diagnosis is critical for preventing unnecessary complications, currently available tests do not exhibit sufficient diagnostic sensitivity in the beginning of disease. We took advantage of high throughput techniques to perform an embracing study of the humoral immune response to the bacteria in order to identify antigens that could be used in a new test for the diagnosis of leptospirosis. A protein microarray chip containing 2,241 leptospiral proteins was constructed and probed with serum samples from patients and healthy individuals. We identified 24 proteins that are recognized by patients' sera but not by healthy individuals. These proteins are potential diagnostic markers, especially the ones identified for acute-phase patients, which can discriminate between a positive and a negative leptospirosis case within a few days after onset of symptoms. This work establishes the protein microarray approach for improving our understanding of the serological response to leptospirosis. Further research is needed to assess the performance of these antigens in the clinical setting.

## Introduction

Leptospirosis is one of the most common zoonotic infectious diseases worldwide. Humans usually become infected through occupational, recreational or domestic contact with the urine of reservoir animals, either directly or through contaminated soil or water [1]–[3]. Pathogenic leptospires frequently produce an asymptomatic infection in wild rodents and other reservoirs; however, in humans and other accidental hosts, it can cause hepato-renal failure, pulmonary hemorrhage syndrome and even death depending on bacterial virulence and the host immune response [1], [2]. Such complications can be prevented if the proper antibiotic therapy is initiated at the onset of the disease [3]–[6]. Nevertheless, the lack of a rapid and reliable diagnostic test is a major barrier to providing an early diagnosis.

Clinical diagnosis of leptospirosis is hindered by the overlapping clinical manifestations with other febrile illnesses [2], [4], [7]. Therefore, the diagnosis depends upon laboratory tests and different methods have been developed. Recovery of leptospires from clinical specimens such as tissue and blood by culture is considered a definitive diagnosis. This diagnosis is hampered, however, by the slow growth rate, the long incubation period until culture is established, low sensitivity and high cost due to the human and laboratory resources required [2]. Nucleic acid-based amplification techniques to detecting leptospiral DNA in biological specimens have also been developed but sensitivity usually decreases as patient progresses to the late stages of disease [2], [4]. Hence, serology is the most frequently used diagnostic approach for leptospirosis.

The gold standard assay is the microagglutination test (MAT), which may use a panel of 19 live leptospires representing the major serogroups for the detection of agglutinating antibodies [2]–[4], [7], [8]. Despite the high specificity, the MAT usually requires paired acute- and convalescent-phase samples, thus being insensitive in the beginning of the disease. To overcome the drawbacks of MAT, numerous serological assays have been developed, particularly ELISA tests based on either whole cell extracts or recombinant proteins [3], [4], [7]. However, these assays have similar performance characteristics, with sensitivity and specificity rates that match those of MAT. Among other serological approaches whose accuracy has been described are agglutination, dipstick, and lateral flow assays [7]. Together, these assays demonstrated low sensitivity during acute phase, so the need for an efficient method to diagnose early infection remains urgent.

High-density protein microarrays are an effective approach to perform large scale serological studies and define antigen-specific antibody responses to infectious agents on a whole proteome scale. They can be produced and probed in a high-throughput manner, allowing for the screening of hundreds of serum samples thus improving the statistical power and generating more accurate conclusions. Additionally, unlike cell extracts, a set of antigens can be identified with optimal sensitivity and specificity. The aims of this research approach are to understand the breadth, intensity and diversity of the antibody response to leptospirosis disease and to discover novel antigens that can be employed in diagnostic tests and subunit vaccines.

Here we report the results of a study probing more than 250 human serum samples, including healthy controls and leptospirosis cases from the state of Bahia, Brazil, against a partial proteome microarray chip containing 2,421 proteins from *Leptospira interrogans* serovar Copenhageni strain L1–130, which was isolated in Bahia, Brazil in 1996. The reason for choosing this specific strain relies on the availability of *L. interrogans* serovar Copenhageni complete genome sequence. Moreover, leptospirosis is an emerging health problem in developing countries. In Brazil, 4832 laboratory-confirmed cases were reported in 2011, distributed among the North (484 cases), Northeast (890 cases), Southeast (1762 cases), South (1673 cases) and Central-West (23 cases) regions [9]. Our group has shown that urban transmission of leptospirosis in Brazil is related to the presence of domestic rats in the environment [10]–[12]. Accordingly, >90% of the leptospirosis cases there are caused by *L. interrogans* serovar Copenhageni, which is commonly associated with *Rattus* species reservoirs [10], [11]. The homogeneity of pathogen exposure and availability of sequenced genomic material from a related strain makes this clinical setting ideal for an initial proteomic study.

## Materials and Methods

### Ethics statement

The study protocol was approved by the institutional review board committees of Yale University and Oswaldo Cruz Foundation. Samples from infected patients and healthy individuals living in a community with high endemic transmission of leptospirosis came from the following projects: “Epidemic Urban Leptospirosis in Salvador, Brazil: A Study of the Clinical Presentation and Development of Rapid Diagnostic Methods” and “Natural History of Urban Leptospirosis”. The participants involved in both projects provided written informed consent. Blood donors from the city of Salvador were anonymous. Sera from U.S. healthy individuals were obtained from anonymous volunteers at the General Clinical Research Center at the University of California, Irvine. After collection, a code number was designated to each patient so that all samples were rendered anonymized for researchers before its use.

### Human sera samples

The evaluation was performed with a collection of 114 control human serum samples and 160 laboratory-confirmed sera of leptospirosis cases. Control samples were (i) 29 sera from healthy volunteers from California/US, where endemic transmission of leptospirosis does not exist; (ii) 35 sera from blood donors from Salvador/Brazil, city with endemic transmission of leptospirosis and (ii) 50 sera from healthy subjects who were enrolled in a cohort study in a high risk urban slum community in the same city [12]. Cases were identified during active hospital-based surveillance in the same state of the slum community, including patients from the city of Salvador and from the country side, from April 1996 to August 2010. During this period, 1529 MAT-confirmed cases of severe leptospirosis were identified, of which we selected 80 acute- and 80 convalescent-phase sera to conduct this study. Serum samples were randomly selected and therefore acute and convalescent samples are not necessarily paired. Acute-phase samples were collected upon patient admittance at the hospital and convalescent-phase samples were collected from recovering patients at least 14 days after hospital admittance and that may or may not have received standard antibiotic therapy. Laboratory confirmation was defined according to the criteria for seroconversion, a four-fold rise in titer or a single titer of 1∶800 in the MAT.

### Microarray targets' selection

Selection of the ORFs that would compose the array was performed considering the *Leptospira interrogans* serovar Copenhageni strain Fiocruz L1–130 genome annotations available at National Center for Biotechnology Information (NCBI) and at John Craig Venter Institute (JCVI) databases. The criteria used included proteins with potentially biological importance [13], [14] and also with potential antigenic features [15]–[18] (Table S1).

### PCR amplification and high throughput recombination cloning

The selected ORFs were attempted to be amplified by PCR and cloned into pXI vector using a high-throughput PCR recombination cloning method described elsewhere [19]. Briefly, ORFs were amplified using 5 ng of *L. interrogans* serovar Copenhageni strain Fiocruz L1–130 with Accuprime Taq DNA Polimerase System (Invitrogen) according to the manufacturer's protocol. Cycling conditions were as follows: 94°C-2 min, 31 cycles of 94°C-90 s, 55°C-15 s, 50°C-15 s, 68°C-2 min and a final extension of 68°C-10 min. Primers contained a 20 bp ORF-specific sequence and a unique 20 bp “adapter” sequence, which becomes incorporated into the 5′ and 3′ termini flanking the amplified gene and is homologous to the cloning sites of the linearized pXI vector (ACGACAAGCATATGCTCGAG and TCCGGAACATCGTATGGGTA, respectively). Genes larger than 3 kb were cloned as smaller segments, maintaining an overlap of at least 150 nt between the sequences, since high throughput cloning efficiency declines when genes are larger than ∼2,500 bp. The segmented ORFs were named with the gene ID followed by the letter “s” and the number of the segment, e.g. LIC10502-s4. The *ligA* and *ligB* genes (LIC10465 and LIC10464, respectively) were fragmented considering the repeated Big domains present in the proteins' structures (LigB Repeats 7–12, LigA Repeats 7–13 and LigA/B Repeats 1–6) [20], which have been previously described as diagnostic markers and/or vaccine candidates [21]–[24]. Up to 3 additional rounds of amplification were attempted for failures, which were usually recovered by adjusting the PCR conditions. All PCR reactions were confirmed for correct insert size by gel electrophoresis before cloning.

The pXI plasmid encodes an N-terminal 6×His-tag and a C-terminal hemagglutinin (HA) tag. The plasmid was linearized by digestion with *Bam*H1 and amplified by PCR to generate the acceptor vector as described previously [19]. A reaction containing 40 ng of linearized pXI vector, 1 uL of ORF PCR reaction and 10 uL of super-competent *Escherichia coli* DH5-α cells (McLab) was incubated on ice for 30 min, heat-shocked at 42°C for 1 min and chilled on ice for 1 min. One hundred and eighty microliters of S.O.C medium were added and cells were cultured for 1 hour at 37°C. The entire reaction mixture was added to 1.1 mL of LB supplemented with kanamycin 50 ug/mL and incubated overnight at 37°C with vigorous aeration. Plasmids were extracted with QIAprep 96 Turbo Kit (Qiagen) without colony selection and analyzed by gel electrophoresis to confirm insert size. Up to 2 additional rounds of cloning were performed to increase efficiency and were resumed by doubling the PCR volume for transformation. All plasmids carrying inserts <500 bp and some randomly selected ones were confirmed for insert presence by PCR using the insert specific primers. After probing the microarrays with the serum samples, the seroreactive antigens were identified and the corresponding plasmids were sequenced. The insert was confirmed in all cases.

### Microarray fabrication and probing

For array fabrication, purified minipreparations of DNA were used for expression in an *E. coli* based *in vitro* transcription-translation (IVTT) reaction system (RTS Kit, Roche) according to the manufacturer's instructions. Ten-microliter reactions were performed in 384-well plates and incubated for 16 hours at 26°C under 300 rpm shaking. Control reactions were performed in the absence of DNA (“NoDNA” controls) to assess the background given by the IVTT reaction itself. Protease inhibitor mixture (Complete, Roche) and Tween-20 to a final concentration of 0.5% v/v were added to the reactions, which were then mixed and centrifuged to pellet any precipitates and remove bubbles prior to printing. Unpurified supernatants were immediately printed onto nitrocellulose coated glass FAST slides using an Omni Grid 100 microarray printer (Genomic Solutions). In addition, arrays were printed with multiple negative control reactions, positive control spots of an IgG mix containing mouse, rat and human IgGs (Jackson ImmunoResearch) and purified Epstein-Barr Virus Nuclear Antigen 1 (EBNA1) protein, which is recognized by the majority of humans thus serving as a marker for serum quality.

Protein expression was verified by probing the array with monoclonal anti-polyhistidine (Sigma Aldrich) and anti-hemaglutinin (Roche Applied Science) against the respective tags. First, arrays were blocked for 30 min with Protein Array Blocking Buffer (Whatman) and probed overnight with anti-tag antibodies diluted 1/400 in Blocking Buffer. Arrays were then incubated for one hour in biotinylated secondary antibodies (Jackson ImmunoResearch) diluted 1/1000 followed by one-hour incubation with streptavidin-conjugated SureLight P3 (Columbia Biosciences). After each incubation, slides were washed 3 times with Tris-buffered saline containing Tween-20 0.05% v/v (TTBS). Additional washes with TBS and distilled water were performed and the slides were air-dried by brief centrifugation before scanning. Slides were scanned in a Perkin Elmer ScanArray confocal laser and intensities were quantified using QuantArray package.

For probing with human serum, samples were diluted 1/100 in Protein Array Blocking Buffer containing *E. coli* lysate 10 mg/mL (McLab) at a final concentration of 10% v/v and incubated for 30 min at room temperature under constant mixing to remove background reactivity to *E. coli* proteins in the IVTT reactions. *E. coli* protein-antibody complexes were removed from the sample dilution mix via centrifugation prior to addition to the microarray. Arrays were blocked for 30 min with Protein Array Blocking Buffer and then incubated with diluted samples overnight at 4°C, with gentle rocking. Biotinylated anti-human immunoglobulin G (Fc-γ fragment specific, Jackson ImmunoResearch) was diluted 1/2000 in Blocking Buffer and added to the arrays for one-hour incubation at room temperature. Slides were washed 3 times with TTBS after each incubation and bound antibodies were detected by one-hour incubation with streptavidin-conjugated SureLight P3, as described above. Finally, slides were scanned for intensity quantification.

### Immunostrips probing

Eleven clones, corresponding to the 10 most differentially reactive antigens for either acute or convalescent groups (see results), were submitted to a five-hour IVTT reaction (RTS, Roche) according to the manufacturer's instructions. Protease inhibitor mixture (Complete, Roche), Tween-20 and methanol were added to final concentrations of 0.5% and 10% v/v respectively. The reactions were mixed and centrifuged to remove bubbles. Unpurified supernatants were printed on Hi-Flow Plus HF240 membrane (Millipore) using a BioJet dispenser (BioDot) at 1 uL/cm and cut into 3 mm strips. Individual strips were then blocked in TTBS 5% non-fat milk for 30 min. Sera samples were diluted 1/250 in TTBS 5% nonfat milk containing *E. coli* lysate at a final concentration of 20% v/v and incubated for 30 min at room temperature under agitation. Blocked strips were then incubated with diluted sera during 1 hour and washed 6 times with TTBS. Alkaline phosphatase-conjugated anti-human IgG (Jackson ImmunoResearch) was diluted 1/5000 in TTBS 5% nonfat milk and applied to each strip for 1 hour at room temperature under agitation. After washing 6 times with TTBS, 3 additional washes with TBS were performed and reactive bands were visualized by incubation with 1-step Nitro-Blue Tetrazolium Chloride/5-Bromo-4-Chloro-3′-Indolyphosphate p-Toluidine Salt (NBT/BCIP) developing buffer (Thermo Fisher Scientific) for 2 min at room temperature. Enzymatic reaction was stopped with tap water and the strips were air-dried before scanning at 2,400 dpi (Hewlett-Parckard scanner). Images were converted to gray scale and band intensities were quantified using the ImageJ software (found at http://rsbweb.nih.gov/ij/).

### Data analysis

Spot intensities were quantified using QuantArray software. Raw data were obtained as the mean pixel signal intensity for each spot and all intensities were automatically corrected for spot-specific background. For each array, the average of control IVTT reactions (NoDNA controls) was subtracted from spots' signal intensities in order to minimize background reactivity. Proteins were considered to be expressed when signal intensity for either tags was above the NoDNA control reactions mean plus 2.5 standard deviations. The same cut-off was applied to identify the reactive proteins using the sera collection. Data analysis was performed using the R statistical software (found at http://www.r-project.org). To stabilize the variance, VSN normalization was applied to the raw data and groups were compared by a Bayes regularized *t* test adapted from Cyber-T for protein arrays [25], [26]. Benjamini and Hochberg (BH) method was used to control the false discovery rate [27] so that *p*-value smaller than 0.05 was considered significant and the corresponding protein was considered differentially reactive. For plotting the histogram, BH corrected *p*-values smaller than 1E-14 were assigned as 1E-16. Multiplex classifiers were generated using linear and nonlinear Support Vector Machines (SVMs) using the “e1071” R package. SVM is a supervised learning method that has been successfully applied to microarray data characterized by small samples sizes and a large number of attributes. The SVM approach, as any other supervised classification approach, uses a training dataset to build a classification model and a testing set to validate the model. To generate unbiased training and testing sets, leave one out cross-validation (LOOCV) was used. With this methodology, each data point is tested with a classifier trained using all of the remaining data points. Plots of receiver operating characteristic (ROC) curves were made with the “ROCR” R package. Sensitivity and specificity were determined from the resulting ROC curves. Clinical characteristics of the leptospirosis patients whose acute and/or convalescent serum samples were selected for this study were described using frequencies and medians with interquartile (IQR) ranges (Table S2). The Chi square test or the Mann-Whitney/Wilcoxon test was used to compare clinical presentations of acute-phase leptospirosis patients with convalescent-phase patients. An association between patients' clinical characteristics and the intensity of acute sera signal against the three antigens that presented the best performance in the protein microarray were evaluated by the Kruskal-Wallis test.

### Microarray data accession number

The raw and normalized array data used in this study have been deposited in the Gene Expression Omnibus archive (http://www.dtd.nlm.nih.gov/geo/), accession number GSE42720.

## Results

### Protein microarray antigen selection

Characterization of the serological response to Leptospira exposure and infection on a whole proteome scale with protein microarrays has not been previously done. To evaluate the feasibility of this approach for leptospirosis, we identified a subset of proteome more likely to be immunoreactive. The selection criteria used to choose the proteins included in the array provided 2,241 ORFs, which corresponded to 61% of *Leptospira interrogans* proteome. The basis for selecting this particular subset of proteins took advantage of empirical mass spectrometry and RNA expression data available for *Leptospira interogans* [13], [14] and also from proteome microarray data from other Gram negative bacteria [17], [18] (Supplementary Table S1).

In total, the array contained 2361 antigens, including full length proteins and protein segments. Protein expression was evaluated by probing the array with anti-His and anti-HA, and over 97% of protein spots were confirmed positive for either His or HA tags (Figure S1A).

### Human IgG antibody profile

Sera used in this study were classified into 5 groups, summarized in Table 1 and described in the methods section. Table S2 shows the clinical characteristics of the leptospirosis patients who provided sera for this study. The majority of them (88%) were male and the median age was 34 (IQR: 24–45) years old. Median duration of symptoms before hospitalization was 6 (IQR: 5–8) days. Jaundice and acute respiratory distress syndrome occurred in 87% and 13% of the patients, respectively. Renal impairment was frequent (median creatinine: 4.0 [IQR: 2.0–6.4] mg/dL) and 30% of the patients received peritoneal or hemodialysis. Intensive care was provided for 20% of the patients and 3% died.

**Table 1:** Sera collection used in this study.

Group	# sera probed	MAT median titer	MAT titer range*
U.S. volunteers	29	NA	NA
Blood donors from endemic area	35	NA	NA
Healthy individuals from highly endemic area	50	0	0
Acute phase patients	80	800	0–12,800
Convalescent phase patients	80	3200	0–204,800

NA = not applicable.

* Acute-phase patients with negative MAT result were diagnosed by seroconversion; convalescent patients with negative MAT result were diagnosed based on the acute MAT titer.

**Figure 1:**
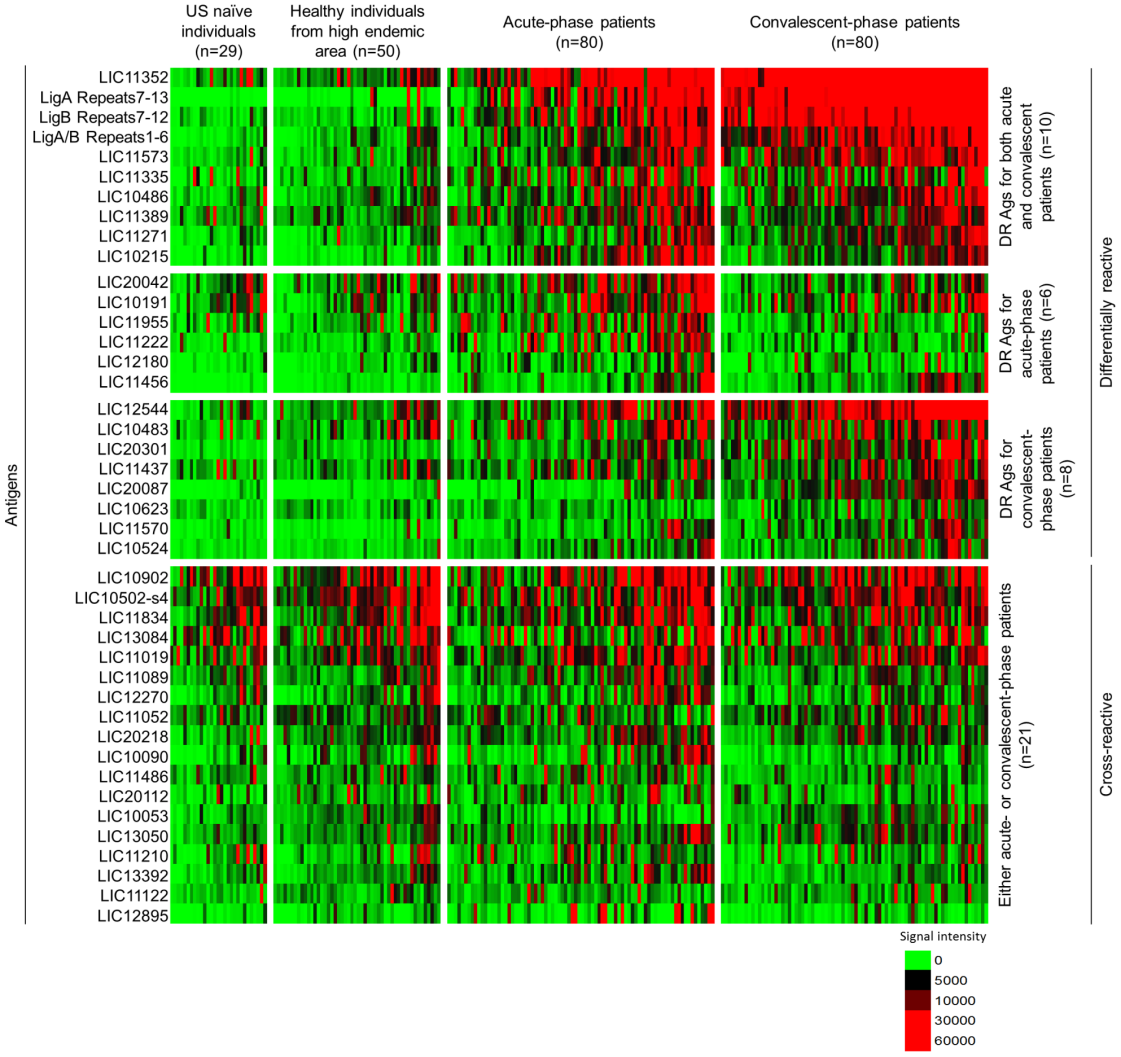
Individual sera are represented as a heatmap of reactivity. Reactivity intensity is shown according to the colorized scale with red strongest, black in-between and green weakest. Antigens are in rows, grouped as differentially reactive (BH*p*<0.05) or cross-reactive (BH*p*>0.05) when compared to healthy individuals from high endemic area group and ranked by the average response of the acute or convalescent groups. Patient samples are in columns and sorted from left to right by increasing average antigen intensity within each group.

Representative microarray images of *L. interrogans* infected and control samples are shown in Figure S1B. The heatmap in Figure 1 gives an overview of the reactivity of the 42 reactive antigens for each of the 239 individual samples. Brazilian blood donors are not shown in this figure. Individual specimens are in columns and grouped by healthy controls from USA, healthy controls from the high endemic area group, acute-phase patients and convalescent-phase patients. The antigens, in rows, are organized according to those that are significantly more reactive in the cases than in the healthy controls. These antigens are termed ‘differentially reactive’ (DR) and are divided in 3 sections: antigens identified as differentially reactive for both acute- and convalescent-phase patients, antigens identified as differentially reactive only for acute patients and differentially reactive antigens only for convalescent patients. There is a second set of antigens that were equally as reactive in healthy controls and the cases, and these antigens are termed ‘cross-reactive’ (CR). Although there was some reactivity seen in the healthy controls against the differentially reactive antigens, there was more IgG response against these antigens after acute infection, and still more in the convalescent specimens. The background reactivity seen from the cross-reactive antigens was similar between all three groups.

**Figure 2:**
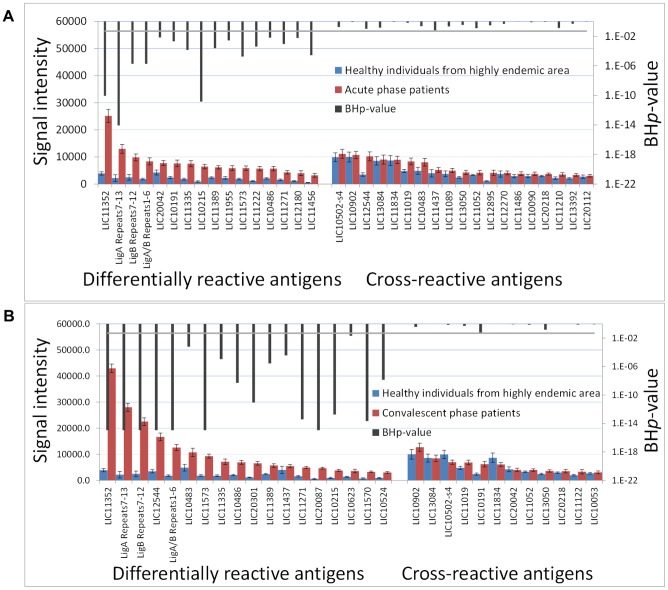
Seroreactive antigens identified for acute- and convalescent-phase patients. The histogram plots the average signal intensity (Y axis) of each antigen (X axis) for acute (A) or convalescent (B) groups against healthy individuals from highly endemic area group, with the BH*p*-value of this difference (black bars, secondary axis). Differentially reactive antigens (BH*p*<0.05) are organized to the left; cross-reactive antigens (BH*p*>0.05) are organized to the right. Error bars indicate S.E. For plotting the histograms, it was assigned the number 10^−16^ for BH*p*-values <1E-14.

**Figure 3:**
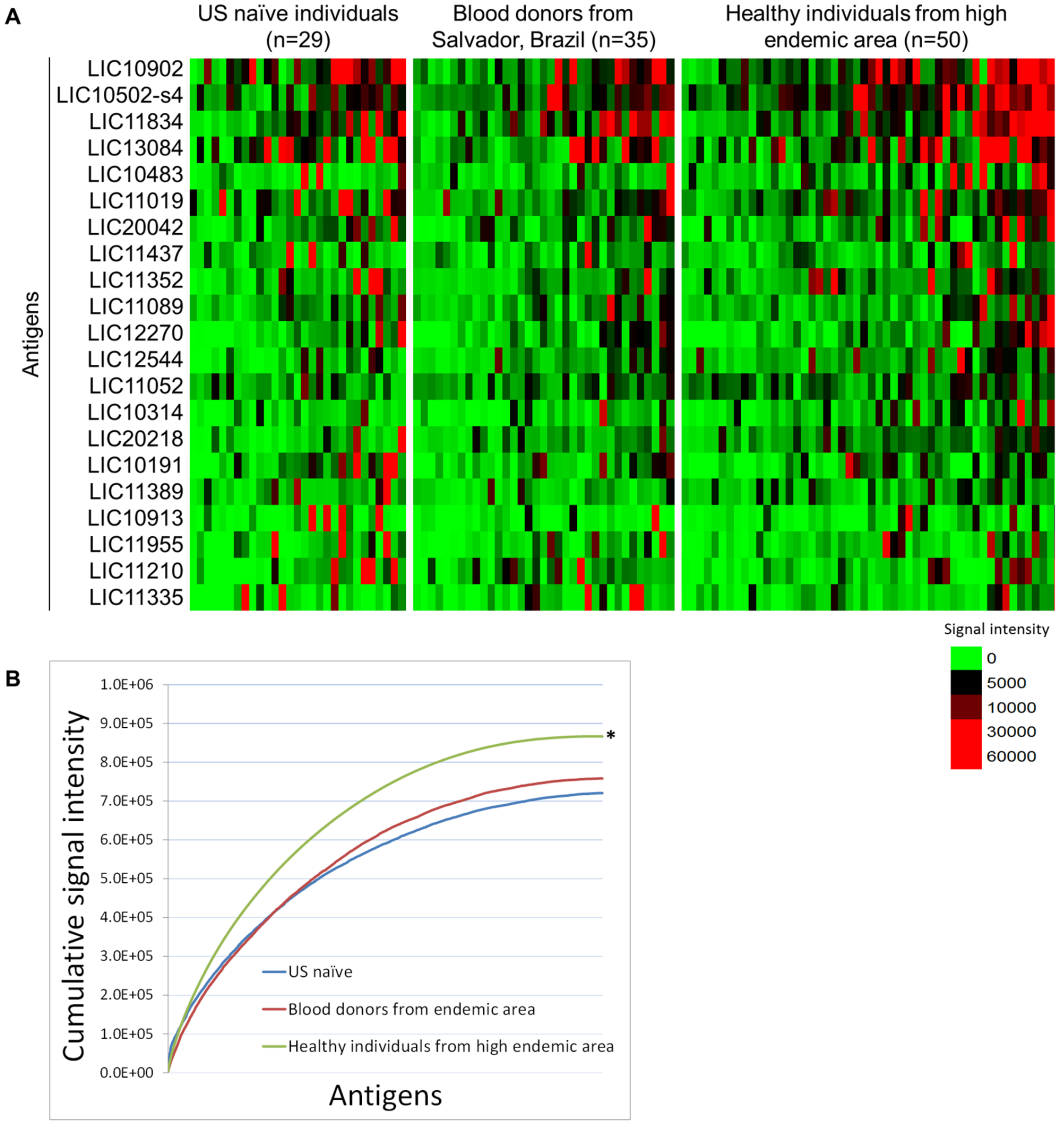
Background reactivity among healthy individuals in the control groups. A) Heatmap showing the overall reactivity of the control groups used in this study: US naïve subject, corresponding to area with no endemic transmission of leptospirosis, blood donors from Salvador, city with endemic transmission and healthy individuals residing in an urban slum community in Salvador, high risk area. B) Cumulative reactivity of all the antigens present in the array for the 3 control groups. Healthy individuals from area with high endemic transmission show significantly higher sero-reactivity (*p*<0.05, marked with a star) than individuals from regions with endemic or non-endemic transmission of leptospirosis.

Here, we aim to identify antigens that can discriminate between positive and negative leptospirosis cases and for that we based our analysis on comparing acute and convalescent-phase patients to healthy individuals from an area with high endemic transmission (Figure 2). Since healthy individuals living in this area show some background reactivity to leptospiral LPS [12] and proteins (Figure 3, described later in this section), we find that the identification of antigens with sero-reactivity among patients but not among those healthy individuals distinguish a current leptospirosis case. All the high endemic controls used in this study were MAT-negative for leptospirosis and in order to avoid bias in our analysis, we compared the IgG reactivity detected on the microarray by probing 10 MAT-positive and 10 MAT-negative healthy individuals living in this area. The overall reactivity seen for both groups was low (Figure S2 A) and most of the reactive antigens detected for infected patients (described later in this section) were not reactive (average signal intensity below the cut-off, Figure S2 B) for either MAT-positive or MAT-negative healthy individuals. Therefore, we used the MAT-negative high endemic controls for the following analysis.

There were 30 reactive antigens, ∼1.3% of all of the antigens printed on the array, of which 18 detected significantly more IgG antibody in the convalescent samples compared to control individuals from the high endemic area group (Table S3). For the acute-phase samples, the IgG antibody response detected 35 seroreactive antigens or 1.5% of the array, of which 16 discriminate between acute and negative cases. LipL32, LigA Repeats 7–13 and LigB Repeats 7–12 antigens were the three most reactive targets on average for both convalescent- and acute-phase groups. Ten differentially reactive antigens overlap between the acute and convalescent groups.

In order to investigate background reactivity among healthy individuals living in an area with endemic transmission of leptospirosis, we compared the cumulative antigen reactivity for the 3 control groups, from USA, Brazilian blood donors and healthy individuals from the high endemic area groups. The heatmap in Figure 3A shows the reactivity of all antigens with average signal intensity above the cut-off for any of the control groups. We observed a higher overall reactivity in the high endemic area group compared to USA controls and Brazilian blood donors. Accordingly, when we analyzed the cumulative signal intensity against all antigens on the array (Figure 3B), USA healthy subjects showed the lowest total reactivity followed by blood donors from Salvador and healthy individuals from high endemic area. Blood donors living in endemic area had slightly higher reactivity than USA naïve subjects, but the difference was not statistically significant. However, the total background reactivity in healthy individuals residing in the area with high endemic transmission was significantly greater (*p*<0.05) than either the blood donors from Brazil or the USA controls.

Finally, we compared the average signal intensity of all the reactive antigens for each patient to the patient's MAT titer. MAT is based primarily on agglutinating antibodies that bind to leptospiral LPS [28], [29] and does not differentiate between IgM and IgG subtypes. All acute and convalescent samples used in this study were laboratory confirmed for infection by MAT and we observed a 3-fold increase in the median titer for convalescent samples compared to the acute group (from 800 to 3,200, Table 1). Although we have also observed a general increase in antigen signal intensities for the convalescent group compared to acute group (Figure 1 and Figure 2), we were unable to draw a correlation between these two approaches (Figure S3) indicating that MAT antigen and protein antigens identify different antibody pools in these patients.

### Serodiagnostic classifier construction by ROC analysis

**Figure 4:**
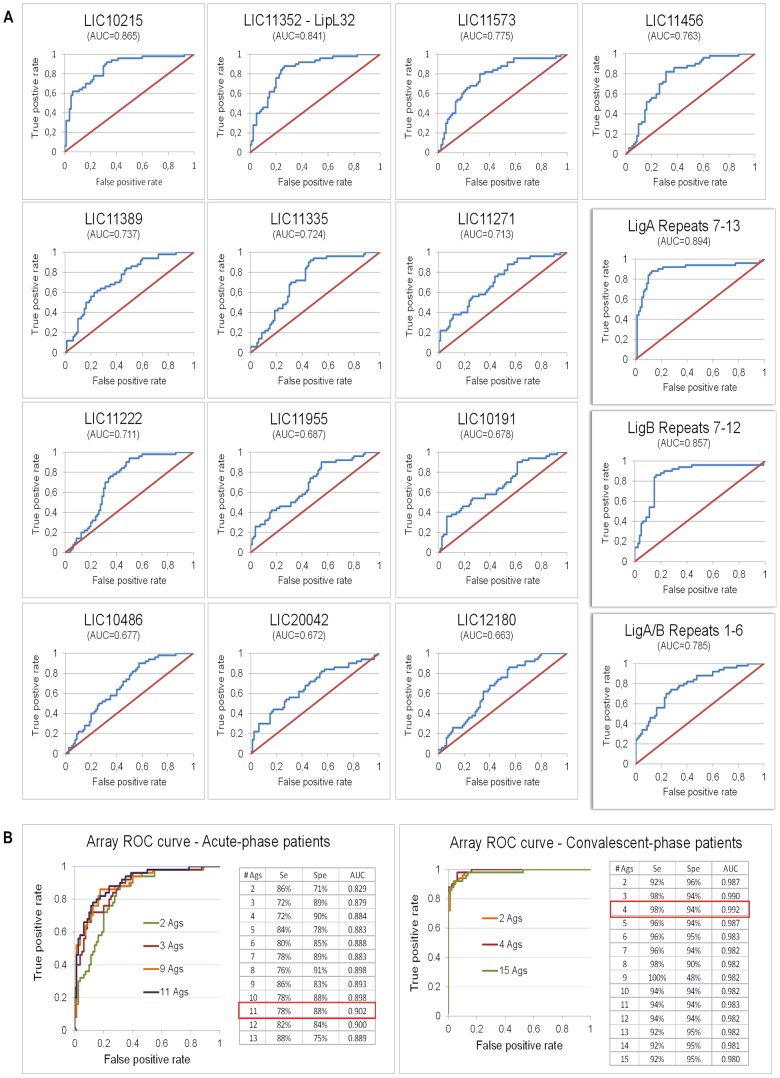
Receiver operator characteristics (ROC) curves. (A) ROC curve for each differentially reactive antigen identified for acute-phase patients when compared to high endemic area group. The domains of the Lig proteins are highlighted on the right; antigens on the left are sorted by decreasing AUC, from left to right, top to bottom. (B) Multiple antigens ROC graphs for acute- (left) and convalescent- (right) phase patients when increasing the number of differentially reactive antigens. Differentially reactive antigens were sorted by decreasing single antigen AUC.

To determine the accuracy of the differentially reactive antigens in distinguishing a leptospirosis case, individual antigen ROC curves were generated and the AUC for each antigen was determined. Acute and convalescent-phase samples were analyzed separately against the high endemic area control group and sensitivity and specificity were calculated for both groups using the SVM computational approach. Antigens were then ranked by decreasing AUC and multiple antigens ROC curves generated. Single antigen ROCs for acute-phase group are shown in Figure 4A and for convalescent-phase group are shown in Figure S4. For both cases, the false positive rate was calculated considering the high endemic area healthy control group.

For acute-phase patients, the non-identical domains of the Lig proteins (LigA Repeats 7–13 and LigB Repeats 7–12) provided best sensitivity and specificity (AUC = 0.894-0.857), followed by LipL32 (LIC11352, AUC = 0.841, Table 2). As disease progresses to convalescence, the accuracy of these antigens increases so that LipL32 achieves best performance (AUC = 0.986) followed by LigA Repeats 7–13 (AUC = 0.965) and LigB Repeats 7–12 (AUC = 0.968, Figure S4). None of the three antigens with better accuracy (LigA Repeats7–13, LigB Repeats7–12 and LipL32) had the signal intensities from the acute serum sample associated with patients' clinical characteristics (Table S4). A heat shock protein of the GroEL family (LIC11335) was also identified as seroreactive, with high sensitivity for both acute- and convalescent-phase patients (90.0% and 92.0%, respectively) but low specificity (53.8% and 62.5%). DnaK (LIC10524), another heat shock protein, showed seroreactivity for the convalescent group, although we could not detect significant levels of IgG against this antigen in the acute group (average signal intensity below the established cut-off). The virulence-associated protein Loa22 (LIC10191) showed very low sensitivity for acute-phase patients (36.0%) and was considered not seroreactive for the convalescent group. Similarly, the IgG response against LipL31 (LIC11456) was detected only among acute patients, with a diagnostic accuracy of 82% sensitivity and 68.8% specificity.

**Table 2:** Accuracy of the differentially reactive antigens for both acute- and convalescent-phase patients after array probing.

Antigen	Acute				Convalescent			
	Se	Spe	AUC	BH*p*-value	Se	Spe	AUC	BH*p*-value
LigA7-13	88.00%	87.50%	0.894	<1E-14	94.00%	98.80%	0.965	<1E-14
LigB7-12	86.00%	83.80%	0.857	8.54E-11	96.00%	96.30%	0.968	<1E-14
LIC11352	88.00%	73.80%	0.841	8.54E-11	92.00%	97.50%	0.986	<1E-14
LIC10215	92.00%	67.50%	0.865	1.42E-11	86.00%	83.80%	0.879	1.79E-13
LIC11573	80.00%	67.50%	0.775	1.73E-05	88.00%	88.80%	0.926	<1E-14
LIC11456^a^	82.00%	68.80%	0.763	2.69E-05	80.00%	78.80%	0.851	1.57E-09
LigA/B1-6	74.00%	72.50%	0.785	1.80E-06	86.00%	96.30%	0.956	<1E-14
LIC11335	92.00%	53.80%	0.724	1.40E-04	90.00%	62.50%	0.749	1.14E-05
LIC11222^b^	94.00%	50.00%	0.711	4.01E-04	94.00%	30.00%	0.568	4.33E-01
LIC11389	60.00%	77.50%	0.737	2.49E-04	66.00%	77.50%	0.77	2.73E-06
LIC11955^b^	90.00%	45.00%	0.687	2.75E-03	90.00%	20.00%	0.477	9.67E-01
LIC11271	88.00%	46.30%	0.713	9.08E-04	88.00%	78.80%	0.877	3.83E-14
LIC10486	90.00%	42.50%	0.677	6.57E-03	90.00%	67.50%	0.831	5.04E-09
LIC12180^b^	86.00%	45.00%	0.663	5.85E-03	88.00%	27.50%	0.534	5.36E-01
LIC10191^b^	36.00%	93.80%	0.678	2.07E-03	90.00%	43.80%	0.655	5.52E-02
LIC20042^b^	82.00%	45.00%	0.672	7.07E-03	22.00%	91.30%	0.525	8.68E-01
LIC20087^a^	76.00%	60.00%	0.699	3.32E-03	96.00%	86.30%	0.948	<1E-14
LIC12544^b^	88.00%	41.30%	0.615	1.03E-01	88.00%	91.30%	0.917	<1E-14
LIC11570^a^	86.00%	56.30%	0.75	3.15E-04	96.00%	78.80%	0.892	2.21E-14
LIC20301^a^	86.00%	51.30%	0.698	2.77E-03	92.00%	75.00%	0.861	8.83E-12
LIC10524^a^	78.00%	76.30%	0.781	1.59E-05	78.00%	81.30%	0.846	1.40E-08
LIC11437^b^	82.00%	45.00%	0.633	6.36E-02	74.00%	72.50%	0.767	3.77E-05
LIC10483^b^	68.00%	50.00%	0.575	2.31E-01	68.00%	67.50%	0.715	6.23E-04
LIC10623^b^	96.00%	11.30%	0.456	6.05E-01	86.00%	43.80%	0.67	2.19E-02

Se = Sensitivity; Spe = Specificity; AUC = Area under the curve. Antigens *in italic* were considered either not seroreactive (^a^) (average signal intensity below the cut-off) or cross-reactive (^b^) (BH*p*<0.05) for that group but were among the differentially reactive set for the other group. NOTE: Different specificities for acute and convalescent-phase cases are a result of the SVM computational analysis, described in the methods section.

Several novel antigens, for which no seroreactivity has been previously described, were identified in this study. The hypothetical protein LIC10215 provided 92.0% and 86.0% sensitivity and 67.5% and 83.8% specificity for distinguishing healthy from either acute- or convalescent-phase patients, respectively. LIC10215 was the best antigen for distinguishing an acute case from a healthy individual after the domains of the Lig proteins and LipL32. Regarding the convalescent group, LIC20087, antigen annotated as outer membrane protein, provided best accuracy after the domains of the Lig proteins and LipL32, with 96.0% sensitivity and 86.3% specificity (Table 2).

The combination of 11 differentially reactive antigens allowed for best sensitivity and specificity for the acute cases (78.0% and 87.5%, respectively) whereas the combination of 4 antigens provided best accuracy (98.0% sensitivity and 94.0% specificity) for convalescent cases (Figure 4B).

### Array validation with immunostrips

**Figure 5:**
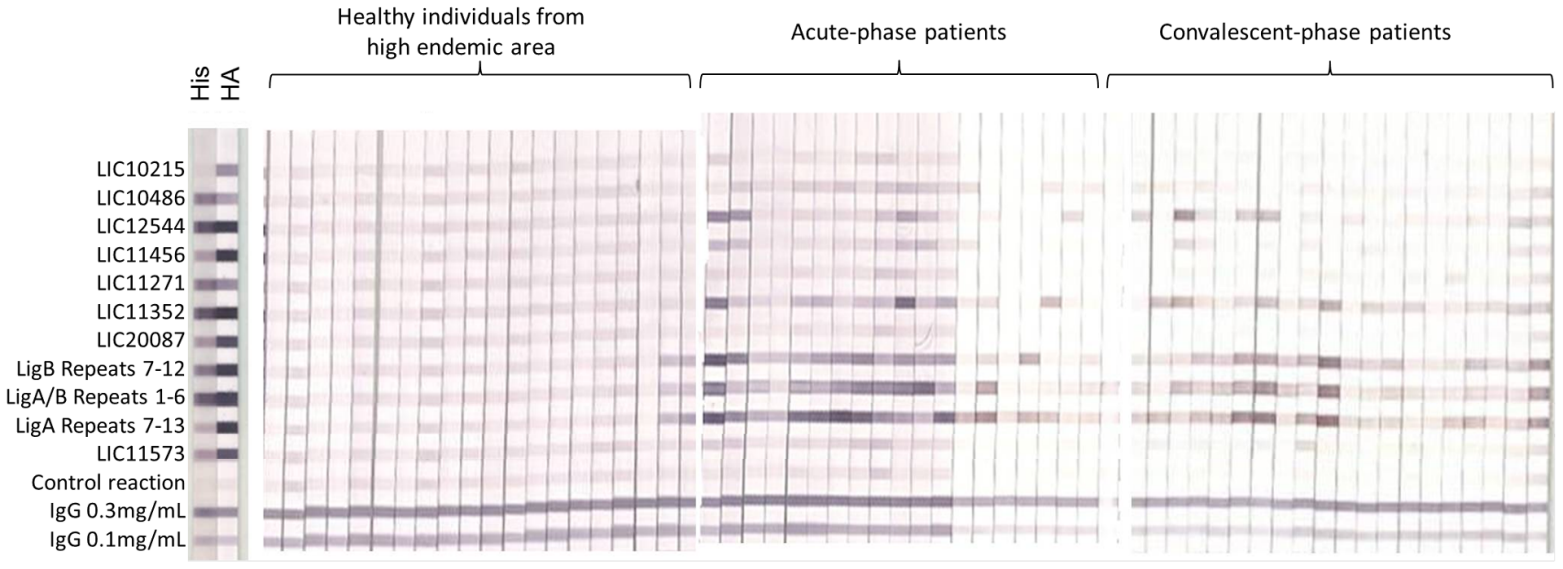
Array validation by immunostrips probing. Strips are grouped by sample type. Probing for His and HA tags are highlighted on the left.

Eleven differentially reactive antigens, corresponding to the most significant antigens for either acute- or convalescent-phase groups were printed onto a nitrocellulose membrane and cut into 3 mm strips which were probed with 20 highly endemic, 20 acute and 20 convalescent randomly selected samples. Healthy individuals showed lower reactivity against these antigens whereas leptospirosis patients reacted strongly against most of the antigens (Figure 5). Antigen intensities were quantified and groups were compared using Bayes regularized *t* test adapted from Cyber-T. A total of 6 antigens with significant BH*p*-values (BH*p*<0.05) were identified as differentially reactive for both acute and convalescent groups, of which 4 overlap (Table S5). For both acute- and convalescent-phase groups, the domains of the Lig proteins provided the best single antigen discrimination, followed by LipL32. LIC10215, LIC10486, LIC11271, LIC20087 and LIC11573 showed no sero-reactivity on immunostrips. The lower reactivity observed for these proteins on immunostrips may be due to technical differences between both platforms.

## Discussion

Protein microarrays are a powerful tool to describe pathogen-specific antibody responses produced after exposure to infectious agents. Our group has applied this approach to more than 25 agents of medical relevance, including viruses, bacteria, protozoan and helminthes and some of the antigens identified by our methodology were successfully employed in different diagnostic platforms [18], [30]–[33]. No currently available approach enables such a complete understanding of the humoral immune response to infection. Here, we constructed a protein microarray comprising 2,421 proteins, 61% of the proteome of *L. interrogans* serovar Copenhageni, to examine the IgG response to leptospirosis. Our focus in the present study was to profile the immune response associated with leptospirosis exposure and infection, and to identify seroreactive and serodiagnostic antigens.

Our results showed distinct IgG reactivity against dozens of differentially reactive leptospiral antigens in both acute- and convalescent-phase sera. The high reactivity detected in most of the acute-phase patients led us to speculate how the IgG response could rise so quickly after infection. The first exposure to an infectious agent in a previously naïve individual is expected to take 10–14 days before mounting an IgG response and the onset of symptoms vary according to the pathogen's incubation period. The incubation period for leptospirosis ranges from as few as 2 to as many as 30 days and the onset of symptoms usually comes together with the appearance of agglutinating antibodies, which increase with disease progression [1]. In this study, the acute-phase patients had a mean of 6 (IQR 5–8) days of symptoms onset before blood sampling and no correlation was observed between IgG reactivity and numbers of days of symptoms before sample collection (Table S4). Therefore, we speculate that the symptomatic individuals with less reactivity in the acute group may have experienced a shorter incubation period before becoming symptomatic compared to those with a broader and more intense response. Alternatively, rapid onset of the IgG responses in acutely infected subjects may be an anamnestic response from a previous clinical or subclinical exposure to the organism. Previously exposed individuals can produce antibody more rapidly from the memory pool within a few days post-exposure.

Here we showed that healthy individuals living in an area with endemic transmission of leptospirosis have higher antibody responses than those from outside the endemic environment. Previous exposure can lead to background reactivity and false positive results, interfering with identification of true active leptospirosis cases especially among those individuals living in areas with endemic transmission. It has been previously reported a 15% overall prevalence of anti-leptospire antibodies detected by MAT in healthy individuals living in that urban slum community (high endemic area group) [12]. Most of the antibodies detected by MAT are directed against leptospiral LPS. Here, we show that MAT-negative healthy individuals living within a community with high endemic transmission of leptospirosis present higher overall seroreactivity against leptospiral proteins than healthy individuals from outside the endemic area, suggesting that protein antigens may also play a role in background reactivity. The shifts in background reactivity between groups of healthy individuals are small compared to the large increases in reactivity seen after acute infection and convalescence. Our results also show that the reactivity against the proteins on the chip doesn't differ between MAT-positive and MAT-negative healthy individuals.

Despite the background reactivity seen for the high endemic area group, we were able to identify several individual antigens that were differentially reactive for acute- and/or convalescent-phase patients when compared to that control group. These antigens can be considered for use alone in single antigen ELISAs or together in a multiplex assay. The diagnostic accuracy was assessed when several antigens were used together in combination. The most accurate test results to distinguish acutely infected subjects from controls were obtained when 11 antigens were combined together and 14 antigens, for convalescent cases. The use of a minimal set of antigens in an assay would represent the best option in terms of production complexity and manufacturing costs. However, our group has previously shown that the addition of antigens can reduce the effect of noise in the data introduced from variables such as executing it in different locations, at different times and by different operators [34]. A multiplex test using several antigens could minimize the effect of these variables and justify the development of a more robust assay of this kind.

Five of the leptospiral proteins identified here have been previously reported reactive in patients' sera including the non-identical domains of the Lig proteins, LipL32, chaperonin GroEL, DnaK and Loa22 [20], [35], [36]. Different platforms have been developed to employ the Lig proteins as serodiagnostic markers for human leptospirosis with promising results [20], [24], [37], [38]. Lig-based immunoblot assays for IgM detection showed superior performance than MAT and superior performance than a commonly used whole-cell ELISA in Brazil during early acute phase [21]. A new Lig-based rapid serological test, the DPP assay, was recently developed and also outperformed the whole-cell IgM ELISA assay for severe acute cases, particularly for patients tested early in the course of the disease [24]. For LipL32, GroEL, DnaK and Loa22, however, the findings were not as encouraging [34], [39], [40], even though LipL32 in combination with LipL21 and OmpL1 [41] improved its diagnostic performance in ELISA platforms. The identification of these previously reported reactive antigens is proof-of-concept for the protein microarray antigen discovery platform.

In this study, the well-known antigens LipL32 and the non-identical domains of the Lig proteins had the best sensitivity and specificity of all antigens probed. The next best differentially reactive antigen for detecting acute-phase patients was the novel hypothetical protein LIC10215. Several other hypothetical proteins also found to be differentially reactive antigens identified in this work were LIC11222, LIC11955, LIC10486, LIC11271, LIC10483 and LIC20301. Although no previous functions have been assigned to these proteins, here we show that they are part of the *L. interrogans* immunoproteome and can elicit a host immune response as they are recognized by sera from infected subjects. We also discovered numerous differentially reactive antigens that are not hypothetical and have been functionally annotated including LIC20042 (BatC), LIC11889 (FlbB), LIC11573 (GspG), LIC12180 (methyltransferase), LIC11456 (LipL31), LIC11437 (adenylate/guanylate cyclase), LIC12544 (DNA binding protein), LIC20087 (outermembrane), LIC10623 (MotB), LIC11570 (GspD).

The results reported here were from a protein microarray derived from one leptospire serovar, *L. interrogans* serovar Copenhageni, probed with sera from acute- and convalescent-phase patients from a well-characterized model epidemiological setting in Salvador [10]–[12]. This study was limited by the restricted number of antigens selected for the array and also by the prevalence of one specific serovar at our study site. Further research is needed to investigate the diversity of the antibody profile after exposure to different serovars. All the samples used here corresponded to hospitalized leptospirosis patients, but the immune response may be different for mild presentations. Finally, we recognize the importance of also evaluating the IgM antibody response to understand the kinetics of the humoral immune response.

In other protein microarray studies of kind we have found that proteins are not randomly selected for recognition by the immune system and antigens share proteomic features that increase their likelihood to be seroreactive and serodiagnostic [15], [16]. Interrogating the antibody response in a whole proteome scale allows molecular features related to antigenicity to be classified. Proteomic feature enrichment analysis for antibody recognition of leptospiral antigens will be the focus of a separate study using the full leptospire proteome consisting of 3,667 proteins, in which we will also assess the IgM reactivity profile to leptospirosis. We also aim to probe with more diverse specimen collections worldwide to better characterize the antibody repertoire against different leptospire species and serovars, and from different mammalian hosts.

In summary, we reported a protein microarray approach for *L. interrogans* serovar Copenhageni and discovered a limited set of 24 differentially reactive antigens. The antigens identified could be applied to improve the accuracy of rapid tests to diagnose leptospirosis in resource-limited settings. The results show that this is a feasible approach that can be applied in the future to study the humoral immune response in other epidemiological settings worldwide, to examine the antibody response after exposure to different leptospire species and determine the antibody profiles elicited by the pathogen in domestic animals and reservoir hosts.

## Supporting Information

Figure S1 **Representative microarray pictures.** (A) Two subarrays showing His (left) and HA (right) probing for protein expression evaluation. Each of the arrays used for this study contained 16 subarrays. Highlighted spots indicate IVTT control reactions (NoDNA, red boxes), IgGmix (orange) and EBNA-1 (green). (B) Representative sub-array showing the difference in the seroreactivity between an individual from high endemic area (negative sample) and a convalescent-phase patient (positive sample).
(TIF)

Figure S2 **Overall IgG response of healthy controls from high endemic area.** (A) IgG response of 10 MAT-positive and 10 MAT-negative endemic controls against 200 antigens is shown as a heatmap of reactivity according to the colorized scale with red strongest, black in-between and green weakest. (B) Average signal intensity of MAT-positive and MAT-negative endemic controls for some of the reactive antigens identified in this study. The green line shows the cut-off and antigens with average signal intensity below that line is not considered significant in this analysis.
(TIF)

Figure S3 **Correlation between MAT assay and array signal intensity.** For each patient, the average signal intensity of the reactive antigens for acute (left) and convalescent (right) phase patients is plotted in the Y axis and the MAT titer in the X axis.
(TIF)

Figure S4 **Receiver operator characteristic curves.** ROC curves for each differentially reactive antigen identified for convalescent-phase patients when compared to high endemic area group. The domains of the Lig proteins are highlighted on the bottom; antigens are sorted by decreasing AUC, from left to right, top to bottom.
(TIF)

Table S1 **List of protein features used for selecting ORFs that would compose the array.**
(DOCX)

Table S2 **Clinical characteristics of the leptospirosis patients providing sera for the protein microarray evaluation.**
(DOCX)

Table S3 **List of seroreactive antigens.**
(DOC)

Table S4 **Reactivity signals of patients' acute serum against the antigens LipL32, LigA Repeats7–13, LigB Repeats7–12 in the protein microarray according to patient characteristics.**
(DOCX)

Table S5 **Accuracy of the differentially reactive antigens after immunostrips probing.**
(DOC)
