# Supplementary figures and images for: Identification of Seroreactive Proteins of Leptospira interrogans Serovar Copenhageni Using a High-Density Protein Microarray Approach
Source: PLoS Negl Trop Dis. 2013 Oct 17;7(10):e2499. doi: 10.1371/journal.pntd.0002499 (PMC3798601; doi:10.1371/journal.pntd.0002499)

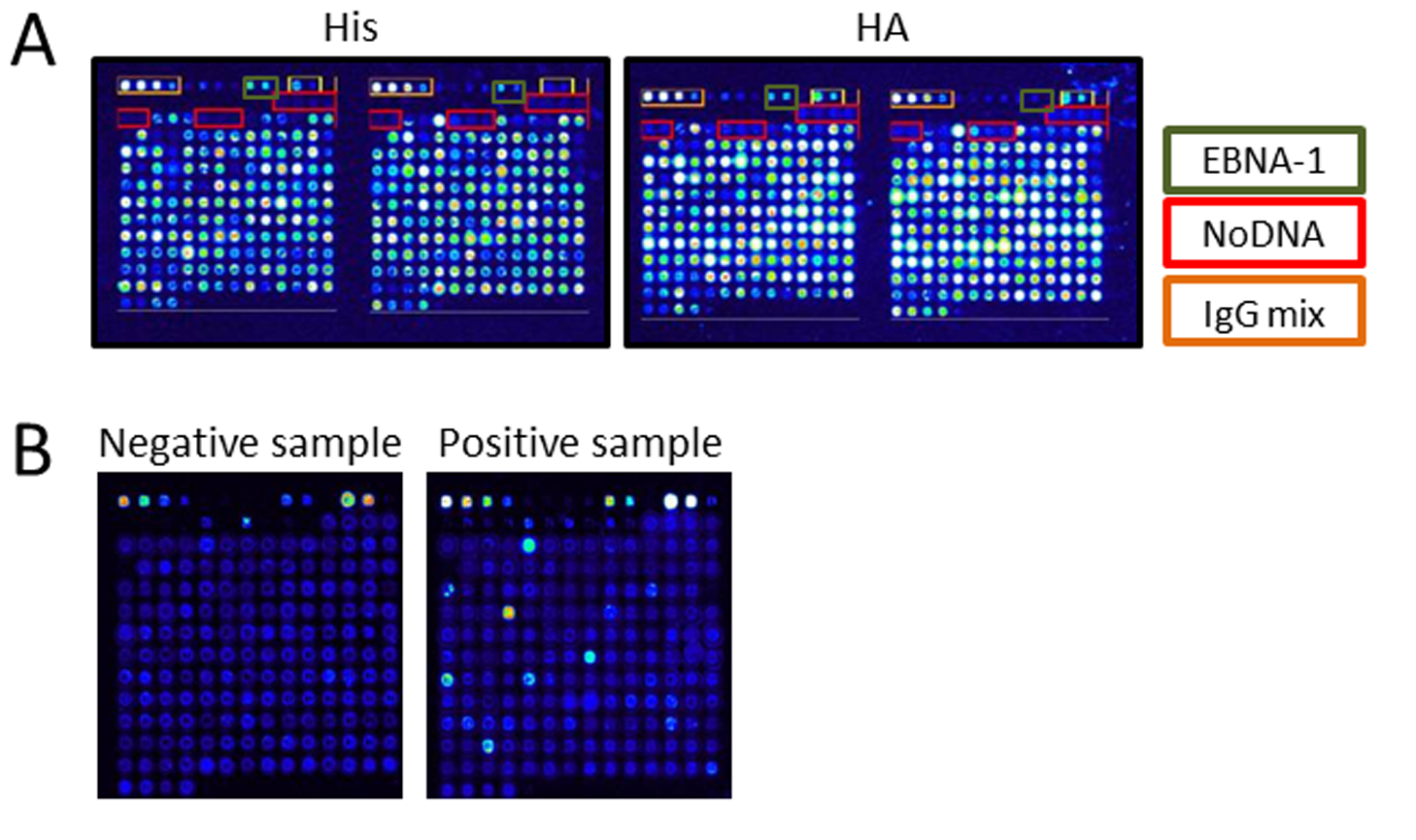

Supplement: Figure S1 — Representative microarray pictures. (A) Two subarrays showing His (left) and HA (right) probing for protein expression evaluation. Each of the arrays used for this study contained 16 subarrays. Highlighted spots indicate IVTT control reactions (NoDNA, red boxes), IgGmix (orange) and EBNA-1 (green). (B) Representative sub-array showing the difference in the seroreactivity between an individual from high endemic area (negative sample) and a convalescent-phase patient (positive sample). (TIF) [file pntd.0002499.s001.tif]

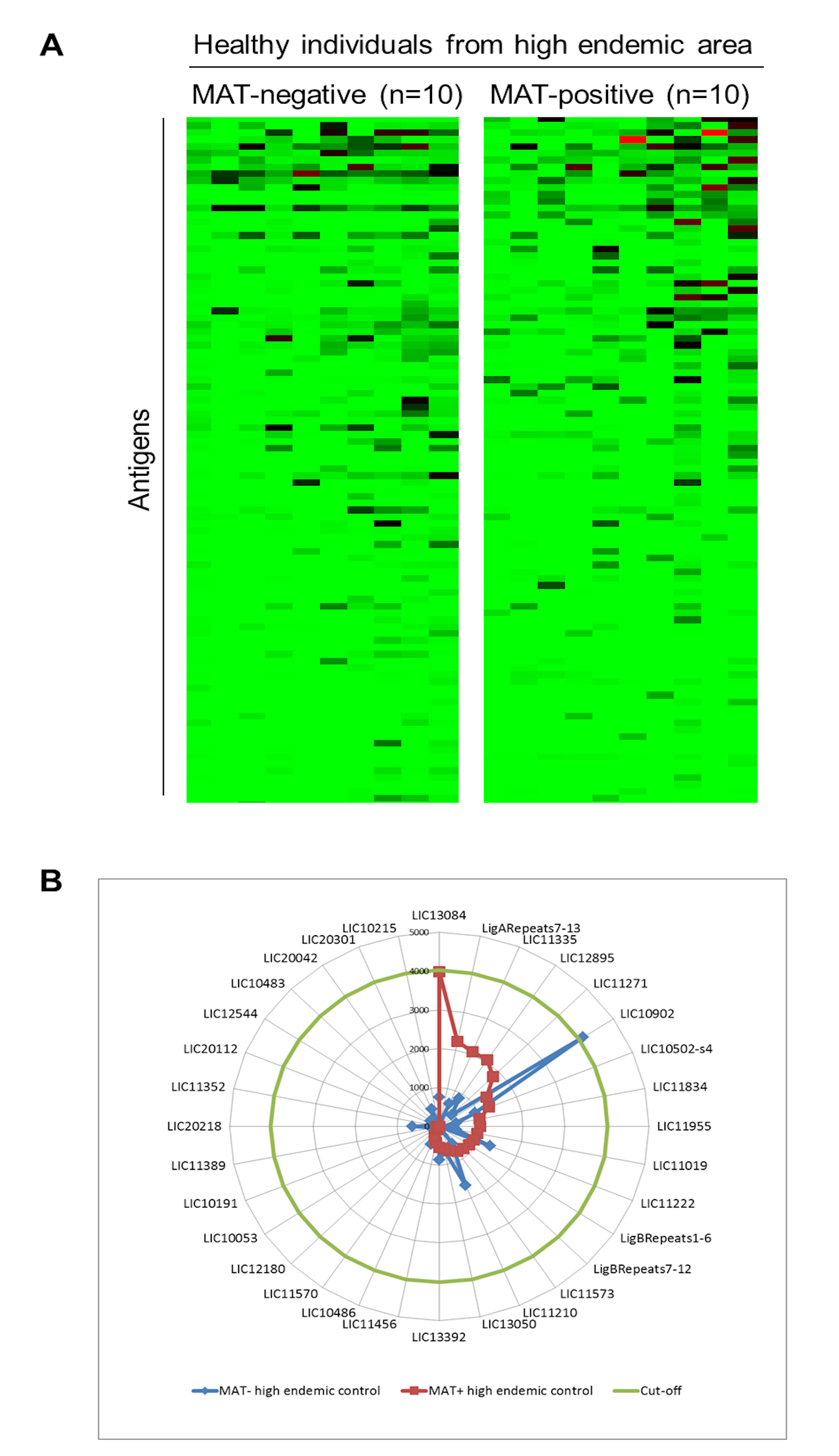

Supplement: Figure S2 — Overall IgG response of healthy controls from high endemic area. (A) IgG response of 10 MAT-positive and 10 MAT-negative endemic controls against 200 antigens is shown as a heatmap of reactivity according to the colorized scale with red strongest, black in-between and green weakest. (B) Average signal intensity of MAT-positive and MAT-negative endemic controls for some of the reactive antigens identified in this study. The green line shows the cut-off and antigens with average signal intensity below that line is not considered significant in this analysis. (TIF) [file pntd.0002499.s002.tif]

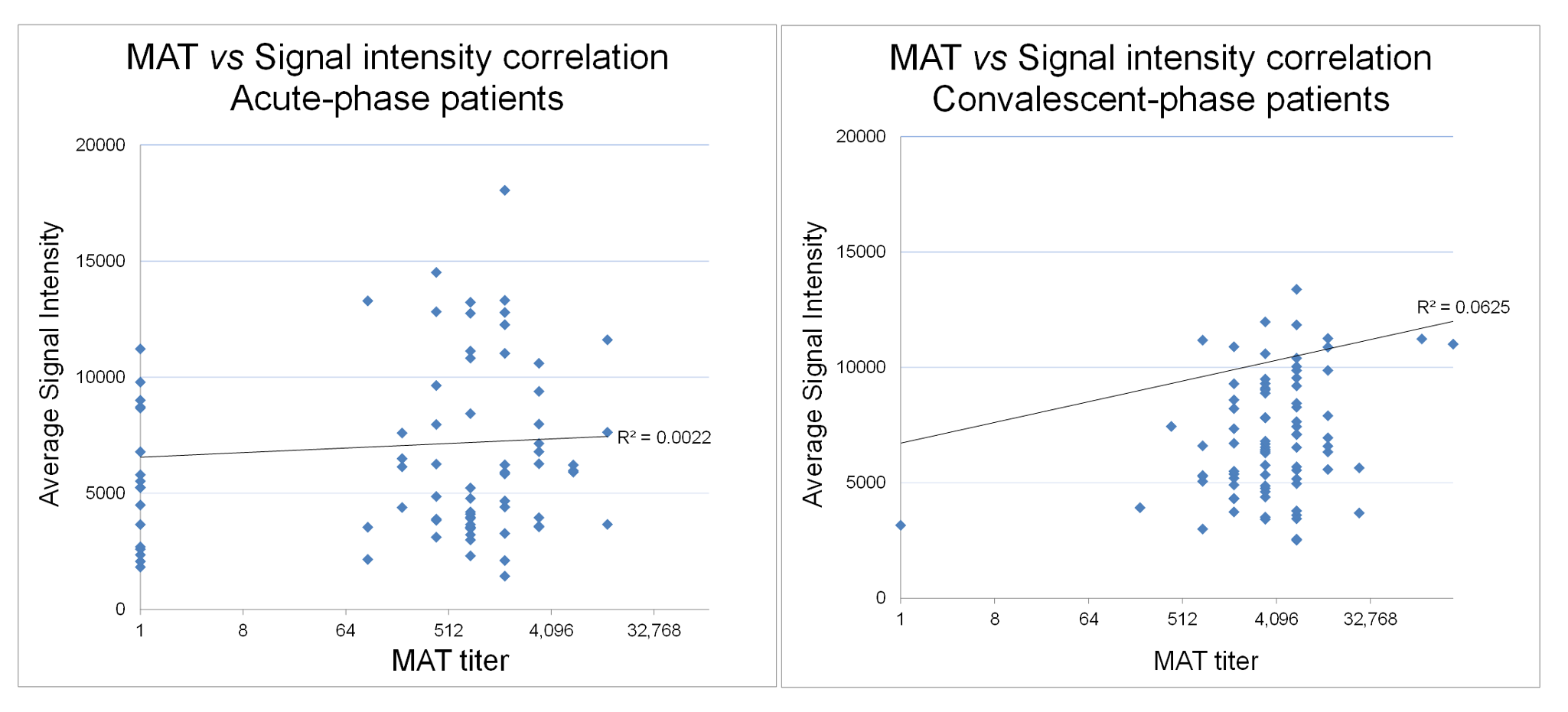

Supplement: Figure S3 — Correlation between MAT assay and array signal intensity. For each patient, the average signal intensity of the reactive antigens for acute (left) and convalescent (right) phase patients is plotted in the Y axis and the MAT titer in the X axis. (TIF) [file pntd.0002499.s003.tif]

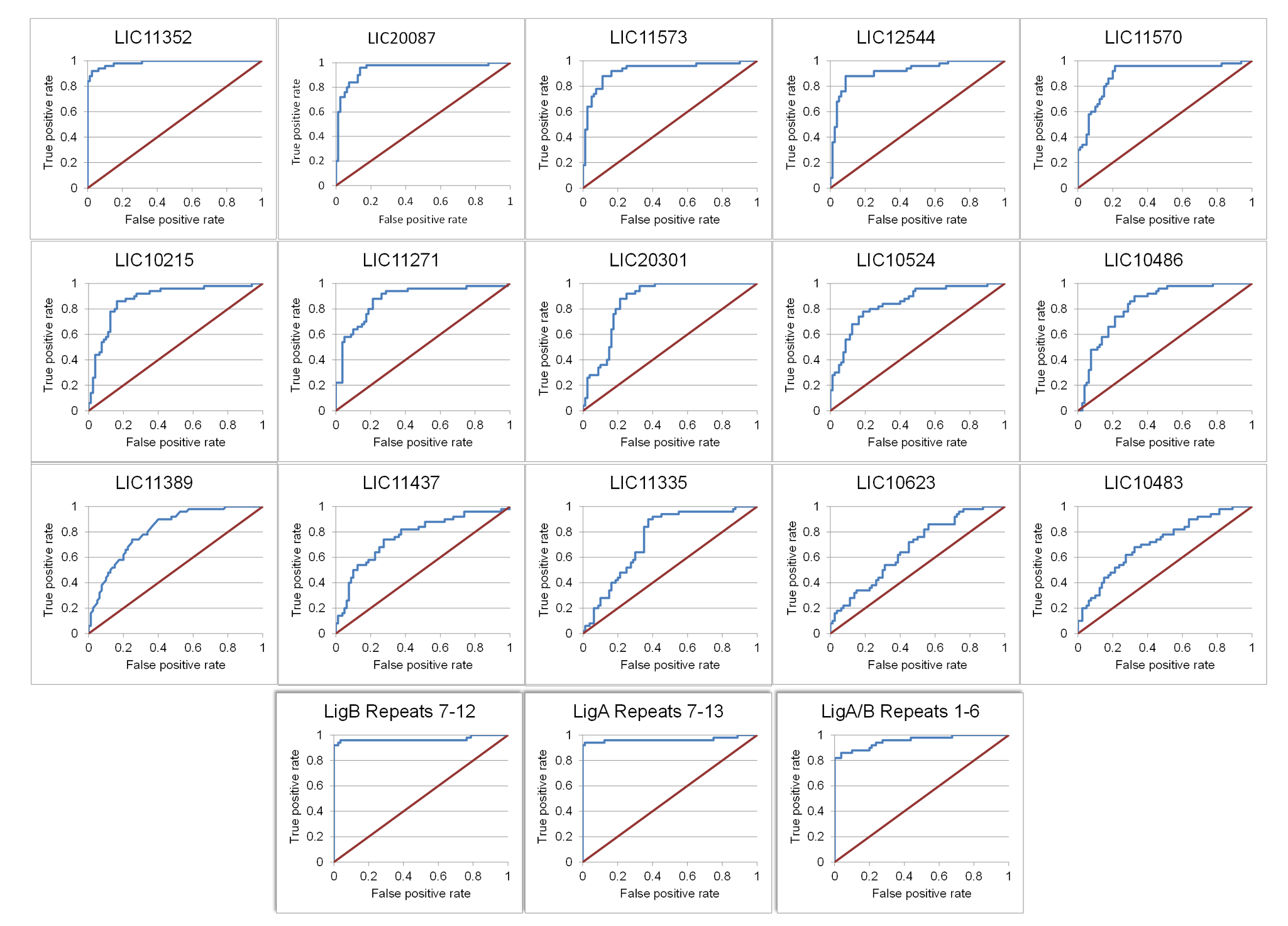

Supplement: Figure S4 — Receiver operator characteristic curves. ROC curves for each differentially reactive antigen identified for convalescent-phase patients when compared to high endemic area group. The domains of the Lig proteins are highlighted on the bottom; antigens are sorted by decreasing AUC, from left to right, top to bottom. (TIF) [file pntd.0002499.s004.tif]
