# Supplementary material for: Identification of Seroreactive Proteins of Leptospira interrogans Serovar Copenhageni Using a High-Density Protein Microarray Approach
Source: PLoS Negl Trop Dis. 2013 Oct 17;7(10):e2499. doi: 10.1371/journal.pntd.0002499 (PMC3798601; doi:10.1371/journal.pntd.0002499)
Supplement: Table S1 — List of protein features used for selecting ORFs that would compose the array. (DOCX) [file pntd.0002499.s005.docx]

| **Category** | **Reference** | **ORF (n)** | **ORF (%)** |
| --- | --- | --- | --- |
| ***Antigenic features*** |  |  |  |
| Signal peptide | SignalP (v3.0, score >0.7, http://www.cbs.dtu.dk/services/SignalP/) | 562 | 25,1 |
| Transmembrane domain | TMHMM (v2.0, http://www.cbs.dtu.dk/services/TMHMM/) | 492 | 22,0 |
| Periplasmic | PSORTb (http://www.psort.org/psortb/) | 29 | 1,3 |
| Outermembrane |  | 62 | 2,8 |
| COG M - Cell wall/membrane biogenesis | NCBI (www.ncbi.nlm.nih.gov/) | 210 | 9,4 |
| COG N - Cell motility |  | 90 | 4,0 |
| COG O - Post translational modification, protein turnover, chaperones |  | 103 | 4,6 |
| COG U - Intracellular trafficking and secretion |  | 72 | 3,2 |
| COG V - Defense mechanisms |  | 38 | 1,7 |
| Cell envelope | JCVI (jcvi.org/) | 325 | 14,5 |
| Transport and binding |  | 136 | 6,1 |
| Fatty acid and phospholipid metabolism |  | 100 | 4,5 |
| Cellular process |  | 144 | 6,4 |
| ***Biological importance*** |  |  |  |
| Up-regulated in physiological osmolarity | [Matsunaga et al. 2007 (10)](file:///E:\UC%20Irvine%20folder\Lepto6%20paper%20draft\Backup%20paper\Tables%20112612.xlsx#RANGE!_ENREF_10) | 121 | 5,4 |
| Positive for mass spectrometry | [Malmstrom et al. 2009 (9)](file:///E:\UC%20Irvine%20folder\Lepto6%20paper%20draft\Backup%20paper\Tables%20112612.xlsx#RANGE!_ENREF_9) | 567 | 25,3 |

NOTE: ORF=Open Reading Frame; ORFs may be included in more than one category.
