# Supplementary material for: Identification of Seroreactive Proteins of Leptospira interrogans Serovar Copenhageni Using a High-Density Protein Microarray Approach
Source: PLoS Negl Trop Dis. 2013 Oct 17;7(10):e2499. doi: 10.1371/journal.pntd.0002499 (PMC3798601; doi:10.1371/journal.pntd.0002499)
Supplement: Table S2 — Clinical characteristics of the leptospirosis patients providing sera for the protein microarray evaluation. (DOCX) [file pntd.0002499.s006.docx]

Table S2. Clinical characteristics of the leptospirosis patients providing sera for the protein microarray evaluation.

| **Characteristics** | **Total patients (N: 157) ^1^** | **Patients with acute sample evaluated (N:80) ^1^** | **Patients with convalescent sample evaluated (N: 80) ^1^** | **P value** |
| --- | --- | --- | --- | --- |
|  | **N (%) or median (IQR)** | | |  |
| **Demographic** |  |  |  |  |
| Male sex | 138 (88) | 67 (84) | 73 (91) | 0.151 |
| Age, years | 34 (25-45) | 34 (27-45) | 34 (24-47) | 0.827 |
| **Clinical** |  |  |  |  |
| Duration of symptoms before hospitalization, days ^2^ | 6 (5-8) | 6 (5-8) | 6 (5-7) | 0.175 |
| Fever ^3^ | 150 (97) | 77 (100) | 76 (95) | 0.120 |
| Conjunctival suffusion ^4^ | 37 (30) | 27 (47) | 12 (17) | <0.001 |
| Jaundice | 137 (87) | 73 (91) | 67 (84) | 0.151 |
| Acute respiratory distress syndrome ^5^ | 21 (13) | 7 (9) | 14 (18) | 0.101 |
| Creatinine, mg/dL ^6^ | 4.0 (2.0-6.4) | 3.5 (2.0-6.1) | 4.6 (2.0-6.5) | 0.446 |
| Urea, mg/dL ^6^ | 142 (97-230) | 146 (98-225) | 141 (99-231) | 0.610 |
| **Outcome** |  |  |  |  |
| Dialysis ^7^ | 45 (30) | 17 (23) | 28 (35) | 0.112 |
| Intensive care unit admission ^6^ | 31 (20) | 16 (20) | 15 (19) | 0.841 |
| Death ^2^ | 5 (3) | 4 (5) | 1 (1) | 0.367 |

IQR=Interquartile range.

^1^ Characteristics for the total patients are presented for 157 individuals because three of them had both acute and convalescent serum sample included in this study. These three patients are included among the group of patients with acute sample evaluated as well as among the group of patients with convalescent sample evaluated.

^2^ Data missing for one patient whose convalescent serum sample were evaluated by the protein microarray.

^3^ Data missing for three patients whose acute serum samples were evaluated by the protein microarray.

^4^ Data missing for 32 patients; 23 and 9 whose acute and convalescent serum samples were evaluated by the protein microarray, respectively.

^5^ Acute respiratory distress syndrome during hospital stay was defined by the presence of respiratory insufficiency (respiratory frequency ≥35 per min or use of mechanical ventilation due to respiratory distress).

^6^ Maximum values during hospital stay.

^7^ Data missing for 7 patients whose acute serum samples were evaluated by the protein microarray.

^8^ Data missing for one patient whose acute serum sample was evaluated by the protein microarray.
