# Supplementary material for: Identification of Seroreactive Proteins of Leptospira interrogans Serovar Copenhageni Using a High-Density Protein Microarray Approach
Source: PLoS Negl Trop Dis. 2013 Oct 17;7(10):e2499. doi: 10.1371/journal.pntd.0002499 (PMC3798601; doi:10.1371/journal.pntd.0002499)
Supplement: Table S3 — List of seroreactive antigens. (DOC) [file pntd.0002499.s007.doc]

Table S3. List of seroreactive antigens.

|  | | |  | Acute group | | | Convalescent group | | |
| --- | --- | --- | --- | --- | --- | --- | --- | --- | --- |
| Antigen ID | | Product | | Average signal intensity | BH*p*-value | Reactivity classification | Average signal intensity | BH*p*-value | Reactivity classification |
| LIC11352 | LipL32 | | | 25150.3 | 8.54E-11 | DR | 42950.1 | 0 | DR |
| LigA Repeats 7-13 | Lig protein A | | | 13002.0 | 8.54E-11 | DR | 27995.7 | 0 | DR |
| LigB Repeats 7-12 | Lig protein B | | | 9824.4 | 1.80E-06 | DR | 22508.6 | 0 | DR |
| LigB Repeats 1-6 | Lig protein B | | | 8351.1 | 0 | DR | 12572.6 | 0 | DR |
| LIC20042 | BatC | | | 7718.2 | 7.07E-03 | DR | 3991.3 | 8.68E-01 | CR |
| LIC10191 | peptidoglycan associated cytoplasmic membrane protein | | | 7585.8 | 2.07E-03 | DR | 6210.0 | 5.52E-02 | CR |
| LIC11335 | chaperonin GroEL | | | 7497.6 | 1.40E-04 | DR | 7122.7 | 1.14E-05 | DR |
| LIC10215 | hypothetical protein LIC10215 | | | 6427.4 | 1.42E-11 | DR | 3804.5 | 1.79E-13 | DR |
| LIC11389 | flagellar protein B | | | 6149.7 | 2.49E-04 | DR | 5692.5 | 2.73E-06 | DR |
| LIC11955 | hypothetical protein LIC11955 | | | 5858.7 | 2.75E-03 | DR | - | - | - |
| LIC11573 | general secretory pathway protein G | | | 5838.1 | 1.73E-05 | DR | 9287.4 | 0 | DR |
| LIC11222 | hypothetical protein LIC11222 | | | 5694.5 | 4.01E-04 | DR | - | - | - |
| LIC10486 | hypothetical protein LIC10486 | | | 5687.5 | 6.57E-03 | DR | 6896.8 | 5.04E-09 | DR |
| LIC11271 | hypothetical protein LIC11271 | | | 4300.9 | 9.08E-04 | DR | 4919.1 | 3.83E-14 | DR |
| LIC12180 | methyltransferase | | | 3982.6 | 5.85E-03 | DR | - | - | - |
| LIC11456 | LipL31 | | | 3191.7 | 2.69E-05 | DR | - | - | - |
| LIC10483 | hypothetical protein LIC10483 | | | 8018.0 | 2.31E-01 | CR | 10707.6 | 6.23E-04 | DR |
| LIC11437 | adenylate/guanylate cyclase | | | 5182.2 | 6.36E-02 | CR | 5434.7 | 3.77E-05 | DR |
| LIC12544 | DNA binding protein | | | 10205.5 | 1.03E-01 | CR | 16651.7 | 0 | DR |
| LIC10502-s4 | cytoplasmic membrane protein | | | 11161.0 | 1.78E-01 | CR | 6979.2 | 7.57E-01 | CR |
| LIC10902 | hypothetical protein LIC10902 | | | 10708.7 | 8.46E-01 | CR | 12698.5 | 3.69E-01 | CR |
| LIC11019 | putative lipoprotein | | | 8317.5 | 6.56E-01 | CR | 6862.7 | 5.45E-01 | CR |
| LIC11052 | hypothetical protein LIC11052 | | | 4182.3 | 1.23E-01 | CR | 3964.1 | 7.84E-01 | CR |
| LIC11834 | putative lipoprotein | | | 8927.6 | 8.95E-01 | CR | 6081.0 | 9.61E-01 | CR |
| LIC13050 | hypothetical protein LIC13050 | | | 4265.3 | 3.45E-01 | CR | 3597.8 | 1.52E-01 | CR |
| LIC13084 | hypothetical protein LIC13084 | | | 9030.7 | 1.44E-01 | CR | 8420.3 | 9.77E-01 | CR |
| LIC20218 | hypothetical protein LIC20218 | | | 3666.6 | 8.94E-01 | CR | 3516.4 | 9.59E-01 | CR |
| LIC10090 | hypothetical protein LIC10090 | | | 3764.8 | 8.11E-01 | CR | - | - | - |
| LIC11089 | hypothetical protein LIC11089 | | | 4943.0 | 2.19E-01 | CR | - | - | - |
| LIC11210 | hypothetical protein LIC11210 | | | 3532.1 | 1.31E-01 | CR | - | - | - |
| LIC11486 | hypothetical protein LIC11486 | | | 3799.1 | 9.71E-01 | CR | - | - | - |
| LIC12270 | hypothetical protein LIC12270 | | | 4089.8 | 4.73E-01 | CR | - | - | - |
| LIC12895 | putative lipoprotein | | | 4117.8 | 2.99E-01 | CR | - | - | - |
| LIC13392 | polysaccharide deacetylase | | | 3339.9 | 4.84E-01 | CR | - | - | - |
| LIC20112 | periplasmic protein, LipL45 homologue | | | 3032.1 | 9.30E-01 | CR | - | - | - |
| LIC20301 | hypothetical protein LIC20301 | | | - | - | - | 6546.6 | 8.83E-12 | DR |
| LIC20087 | outer membrane protein | | | - | - | - | 4660.7 | 0 | DR |
| LIC10623 | flagellar motor protein MotB | | | - | - | - | 3571.0 | 2.19E-02 | DR |
| LIC11570 | general secretory pathway protein D | | | - | - | - | 3233.2 | 2.21E-14 | DR |
| LIC10524 | molecular chaperone DnaK | | | - | - | - | 3054.3 | 1.40E-08 | DR |
| LIC10053 | hypothetical protein LIC10053 | | | - | - | - | 3081.8 | 8.80E-01 | CR |
| LIC11122 | putative lipoprotein | | | - | - | - | 3301.9 | 7.87E-01 | CR |

DR = differentially reactive; CR = cross-reactive when compared to healthy individuals from high endemic area group. Blanks correspond to antigens that were either differentially or cross-reactive for one group but the average signal intensity was below the cut-off for the other group.
