# Supplementary material for: Identification of Seroreactive Proteins of Leptospira interrogans Serovar Copenhageni Using a High-Density Protein Microarray Approach
Source: PLoS Negl Trop Dis. 2013 Oct 17;7(10):e2499. doi: 10.1371/journal.pntd.0002499 (PMC3798601; doi:10.1371/journal.pntd.0002499)
Supplement: Table S4 — Reactivity signals of patients' acute serum against the antigens LipL32, LigA Repeats7–13, LigB Repeats7–12 in the protein microarray according to patient characteristics. (DOCX) [file pntd.0002499.s008.docx]

Table S4. Reactivity signals of patients’ acute serum against the antigens LipL32, LigA Repeats7-13, LigB Repeats7-12 in the protein microarray according to patient characteristics.

| **Characteristic** | **Median (IQR) of the LipL32** **reactivity** | **P value** | **Median (IQR) of the LigA Repeats7-13** **reactivity** | **P value** | **Median (IQR) of the LigB Repeats7-12 reactivity** | **P value** |
| --- | --- | --- | --- | --- | --- | --- |
| **Demographic** |  |  |  |  |  |  |
| Sex |  |  |  |  |  |  |
| Male (n: 67) | 19,897 (3,918-51,237) | 0.461 | 9,219 (2,107-16,875) | 0.667 | 5,388 (2,355-13,600) | 0.974 |
| Female (n: 13) | 11,249 (5,522-16,657) |  | 11,176 (4,251-15,949) |  | 4,660 (2,700-8,566) |  |
| Age groups, years |  |  |  |  |  |  |
| ≤25 (n: 18) | 13,232 (2,468-23,786) | 0.379 | 6,807 (280-14,012) | 0.705 | 8,154 (4,197-13,387) | 0.525 |
| 26-35 (n: 29) | 39,914 (4,775-53,173) |  | 8,936 (2,267-18,022) |  | 4,913 (2,321-13,600) |  |
| 36-45 (n: 16) | 11,317 (5,495-50,703) |  | 9,461 (2,836-12,265) |  | 4,167 (2,009-8,281) |  |
| ≥46 (n: 17) | 19,663 (5,522-46,793) |  | 11,549 (4,618-20,700) |  | 6,605 (2,700-14,256) |  |
| **Clinical** |  |  |  |  |  |  |
| Duration of symptoms before hospitalization, days |  |  |  |  |  |  |
| ≤3 (n: 10) | 52,559 (8,240-57,015) | 0.194 | 12,778 (2,847-20,700) | 0.366 | 11,623 (6,637-19,671) | 0.050 |
| 4-5 (n: 17) | 7,441 (2,985-40,184) |  | 4,618 (535-12,755) |  | 2,355 (1,585-6,400) |  |
| 6-7 (n: 25) | 19,897 (8,785-46,437) |  | 9,702 (3,770-14,403) |  | 4,660 (3,201-12,604) |  |
| ≥8 (n: 28) | 19,134 (6,362-42,441) |  | 11,005 (3,586-16,303) |  | 6,210 (2,796-13,654) |  |
| Jaundice |  |  |  |  |  |  |
| Yes (n: 73) | 19,503 (5,941-48,785) | 0.295 | 9,702 (2,847-16,657) | 0.759 | 5,815 (2,700-13,387) | 0.176 |
| No (n: 7) | 3,517 (2,479-51,463) |  | 4,618 (1,651-26,763) |  | 2,197 (963-10,565) |  |
| Acute respiratory distress syndrome ^1^ |  |  |  |  |  |  |
| Yes (n: 7) | 4,256 (2,256-51,864) | 0.336 | 6,688 (535-34,624) | 0.851 | 2,700 (208-13,600) | 0.250 |
| No (n: 73) | 19,663 (5,941-48,785) |  | 9,702 (2,847-15,949) |  | 5,815 (2,614-13,379) |  |
| Creatinine, mg/dL ^2^ |  |  |  |  |  |  |
| ≤2.00 (n: 23) | 16657 (3497-54485) | 0.078 | 12628 (5630-20700) | 0.324 | 6637 (2613-13379) | 0.252 |
| 2.01-4.00 (n: 21) | 14923 (4775-46437) |  | 5066 (1192-16657) |  | 4289 (2700-10565) |  |
| 4.01-6.00 (n: 16) | 33551 (11967-55483) |  | 10744 (4066-16191) |  | 7779 (4598-16873) |  |
| ≥6.01 (n: 20) | 12486 (2172-32827) |  | 5134 (1811-12956) |  | 3047 (1390-12276) |  |
| **Outcome** |  |  |  |  |  |  |
| Death |  |  |  |  |  |  |
| Yes (n: 4) | 32699 (10146-52300) | 0.675 | 9317 (2840-22622) | 0.930 | 8447 (3591-168366) | 0.757 |
| No (n: 76) | 18080 (5098-49821) |  | 9461 (2187-16766) |  | 4950 (2611-13383) |  |

IQR=Interquartile range.

^1^ Acute respiratory distress syndrome during hospital stay was defined by the presence of respiratory insufficiency (respiratory frequency ≥35 per min or use of mechanical ventilation due to respiratory distress).

^2^ Maximum values during hospital stay.
