# Supplementary material for: Identification of Seroreactive Proteins of Leptospira interrogans Serovar Copenhageni Using a High-Density Protein Microarray Approach
Source: PLoS Negl Trop Dis. 2013 Oct 17;7(10):e2499. doi: 10.1371/journal.pntd.0002499 (PMC3798601; doi:10.1371/journal.pntd.0002499)
Supplement: Table S5 — Accuracy of the differentially reactive antigens after immunostrips probing. (DOC) [file pntd.0002499.s009.doc]

Table S5: Accuracy of the differentially reactive antigens after immunostrips probing.

|  | Acute | | | | Convalescent | | | |
| --- | --- | --- | --- | --- | --- | --- | --- | --- |
| Antigen | Se | Spe | AUC | BH*p*-value | Se | Spe | AUC | BH*p*-value |
| LigA7-13 | 90.0% | 95.0% | 0.950 | 8.15E-08 | 90.0% | 100.0% | 0.940 | 2.18E-06 |
| LigA/B1-6 | 80.0% | 100.0% | 0.943 | 7.46E-06 | 85.0% | 100.0% | 0.948 | 1.29E-05 |
| LigB7-12 | 85.0% | 90.0% | 0.880 | 3.78E-05 | 90.0% | 100.0% | 0.955 | 1.44E-06 |
| LIC11352 | 100.0% | 55.0% | 0.765 | 3.19E-03 | 95.0% | 85.0% | 0.930 | 1.48E-05 |
| LIC10486* | 100.0% | 55.0% | 0.698 | 4.67E-02 | - | - | - | - |
| LIC12544* | - | - | - | - | 90.0% | 45.0% | 0.685 | 2.19E-02 |

Se = Sensitivity; Spe = Specificity; AUC = Area under the curve. *Blanks correspond to antigens that were differentially reactive for one group but the average signal intensity was below the cut-off for the other group.
